# Supplementary material for: Time-Restricted Eating in Real-World Healthcare Settings: Utilisation and Short-Term Outcomes Evaluation
Source: Nutrients. 2024 Dec 23;16(24):4426. doi: 10.3390/nu16244426 (PMC11677662; doi:10.3390/nu16244426)
Supplement: Supplementary file 1 [file nutrients-16-04426-s001.zip › Supplementary File -TRE utilisation study.pdf]

Supplementary Materials

Time-Restricted Eating in Real-World Healthcare Settings: Utilisation and Short-Term Outcomes Evaluation

Contents

- Page 2; Figure S1: Patients' flowchart during their visit to the clinic and data eligibility for the study
- Page 3; Table S1: Factors Associated with First-visit TRE
- Page 3; Table S2: Factors Associated with all TRE use
- Page 4. Table S3: STROBE Checklist for the study

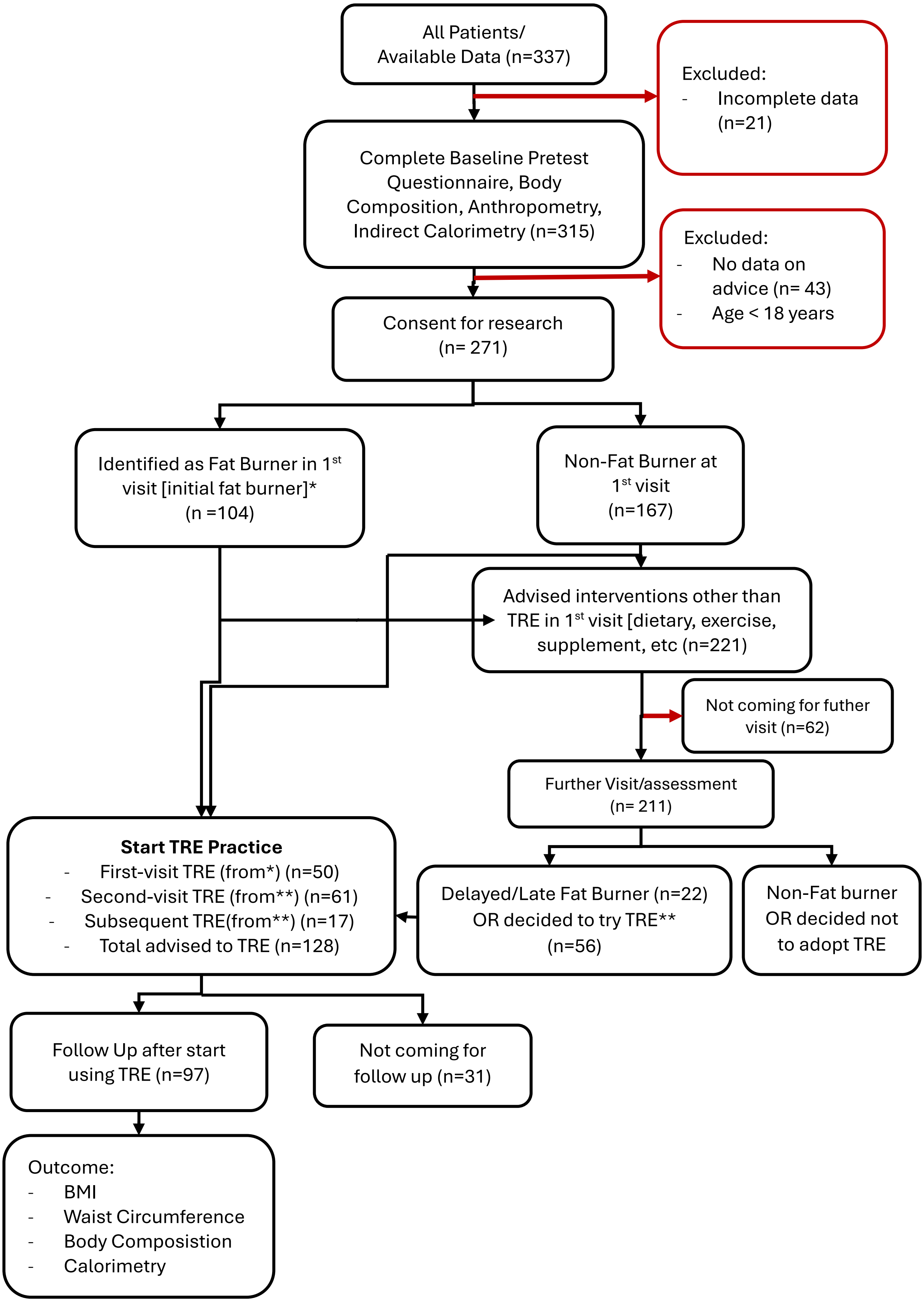

Figure S1. Patients' flowchart during their visits to the clinic

----

Table S1. Factors Associated with First-visit TRE

**Variable**	**adjusted OR**	**(95% CI)**		**p-value**
Respiratory Quotient	23.02631	8.799149	60.25707	0.000*
Preference to low carb	2.359468	0.7740656	7.192014	0.131
Working Status	1.823123	0.959905	3.46261	0.067
Medication Use	1.591637	0.6445564	3.930314	0.314
Age	1.552908	0.6679537	3.610317	0.307
Cardiovascular problem	1.208461	0.5147063	2.837303	0.664
Skipping meals habit	1.112889	0.6970885	1.776706	0.654
Breakfast habit	1.080603	0.7359572	1.586644	0.692
History of Bariatric Surgery	0.7448182	0.1234814	4.492613	0.748
Gender	0.7351123	0.2872381	1.881331	0.521
Baseline BMI	0.7037813	0.3837962	1.29055	0.256
Ethnicity	0.5849809	0.2978812	1.148789	0.119
Diabetes	0.4932531	0.1779566	1.367179	0.174
_cons	0.2861634	0.0036868	22.21162	0.573

Note: *p < 0.05 indicates statistical significance.

Logistic regression	Number of obs	271
	LR chi2(13)	80.19
	Prob > chi2	0.000
Log likelihood = -89.484147	Pseudo R2	0.3094

Table S2. Factors Associated with any TRE timing

**Variable**	**adjusted OR**	**(95% CI)**		**p-value**
Respiratory Quotient	3.105811	1.823349	5.290301	0.000*
Working Status	1.419681	0.9255306	2.177664	0.108
Age	1.27885	0.7394592	2.211693	0.379
Ethnicity	1.274883	0.8319714	1.953584	0.265
Baseline BMI	1.230851	0.782348	1.93647	0.369
Breakfast habit	1.224496	0.9338621	1.60558	0.143
Preference to low carb	1.114351	0.4933223	2.517172	0.795
Skipping meals habit	1.040618	0.7520927	1.439829	0.81
Cardiovascular problem	0.9878197	0.5369351	1.817329	0.969
Gender	0.8905202	0.4690508	1.690704	0.723
Medication Use	0.8815222	0.4897734	1.586614	0.674
Diabetes	0.6098918	0.3171391	1.172886	0.138
History of Bariatric Surgery	0.3599035	0.0858912	1.508077	0.162
_cons	0.0712226	0.0050651	1.001485	0.05

Note: *p < 0.05 indicates statistical significance.

Logistic regression	Number of obs	271
	LR chi2(13)	33.03
	Prob > chi2	0.0017
Log likelihood = -170.9149	Pseudo R2	0.0881

Table S3. STROBE Checklist for the manuscript

		Reporting Item	Page Number
**Title and abstract**			
Title	#1a	Indicate the study's design with a commonly used term in the title or the abstract	1
Abstract	#1b	Provide in the abstract an informative and balanced summary of what was done and what was found	1
**Introduction**			
Background / rationale	#2	Explain the scientific background and rationale for the investigation being reported	2
Objectives	#3	State specific objectives, including any prespecified hypotheses	2
**Methods**			
Study design	#4	Present key elements of study design early in the paper	2
Setting	#5	Describe the setting, locations, and relevant dates, including periods of recruitment, exposure, follow-up, and data collection	2-4
Eligibility criteria	#6a	Give the eligibility criteria, and the sources and methods of selection of participants. Describe methods of follow-up.	4
Eligibility criteria	#6b	For matched studies, give matching criteria and number of exposed and unexposed	supplementary Figure S1
Variables	#7	Clearly define all outcomes, exposures, predictors, potential confounders, and effect modifiers. Give diagnostic criteria, if applicable	4
Data sources / measurement	#8	For each variable of interest give sources of data and details of methods of assessment (measurement). Describe comparability of assessment methods if there is more than one group. Give information separately for for exposed and unexposed groups if applicable.	4
Bias	#9	Describe any efforts to address potential sources of bias	12
Study size	#10	Explain how the study size was arrived at	supplementary Figure S1

Quantitative variables	#11	Explain how quantitative variables were handled in the analyses. If applicable, describe which groupings were chosen, and why	4-5
Statistical methods	#12a	Describe all statistical methods, including those used to control for confounding	4-5
Statistical methods	#12b	Describe any methods used to examine subgroups and interactions	4-5
Statistical methods	#12c	Explain how missing data were addressed	n/a
Statistical methods	#12d	If applicable, explain how loss to follow-up was addressed	n/a
Statistical methods	#12e	Describe any sensitivity analyses	n/a
**Results**			
Participants	#13a	Report numbers of individuals at each stage of study—eg numbers potentially eligible, examined for eligibility, confirmed eligible, included in the study, completing follow-up, and analysed. Give information separately for for exposed and unexposed groups if applicable.	5-6
Participants	#13b	Give reasons for non-participation at each stage	5-6
Participants	#13c	Consider use of a flow diagram	4 and supplementary Figure S1
Descriptive data	#14a	Give characteristics of study participants (eg demographic, clinical, social) and information on exposures and potential confounders. Give information separately for exposed and unexposed groups if applicable.	6-8
Descriptive data	#14b	Indicate number of participants with missing data for each variable of interest	n/a
Descriptive data	#14c	Summarise follow-up time (eg, average and total amount)	8-9
Outcome data	#15	Report numbers of outcome events or summary measures over time. Give information separately for exposed and unexposed groups if applicable.	8-9
Main results	#16a	Give unadjusted estimates and, if applicable, confounder-adjusted estimates and their precision (eg, 95% confidence interval). Make clear which confounders were adjusted for and why they were included	Table 4, page 8-9
Main results	#16b	Report category boundaries when continuous variables were categorized	6-8
Main results	#16c	If relevant, consider translating estimates of relative risk into absolute risk for a meaningful time period	6-8

Other analyses	#17	Report other analyses done—eg analyses of subgroups and interactions, and sensitivity analyses	8
**Discussion**			
Key results	#18	Summarise key results with reference to study objectives	10
Limitations	#19	Discuss limitations of the study, taking into account sources of potential bias or imprecision. Discuss both direction and magnitude of any potential bias.	12
Interpretation	#20	Give a cautious overall interpretation considering objectives, limitations, multiplicity of analyses, results from similar studies, and other relevant evidence.	12
Generalisability	#21	Discuss the generalisability (external validity) of the study results	12
**Other Information**			
Funding	#22	Give the source of funding and the role of the funders for the present study and, if applicable, for the original study on which the present article is based	12
